# Microsensors for *in vivo* Measurement of Glutamate in Brain Tissue

**DOI:** 10.3390/s8116860

**Published:** 2008-11-04

**Authors:** Si Qin, Miranda van der Zeyden, Weite H. Oldenziel, Thomas I.F.H. Cremers, Ben H.C. Westerink

**Affiliations:** Department of Pharmacy, University of Groningen, Deusinglaan 1, 9713AV, Groningen, The Netherlands

**Keywords:** First generation biosensor, second generation biosensor, glutamate, *in vivo* detection

## Abstract

Several immobilized enzyme-based electrochemical biosensors for glutamate detection have been developed over the last decade. In this review, we compare first and second generation sensors. Structures, working mechanisms, interference prevention, *in vitro* detection characteristics and *in vivo* performance are summarized here for those sensors that have successfully detected brain glutamate *in vivo*. In brief, first generation sensors have a simpler structure and are faster in glutamate detection. They also show a better sensitivity to glutamate during calibration *in vitro*. For second generation sensors, besides their less precise detection, their fabrication is difficult to reproduce, even with a semi-automatic dip-coater. Both generations of sensors can detect glutamate levels *in vivo*, but the reported basal levels are different. In general, second generation sensors detect higher basal levels of glutamate compared with the results obtained from first generation sensors. However, whether the detected glutamate is indeed from synaptic sources is an issue that needs further attention.

## 1. Introduction

Glutamate is a major excitatory neurotransmitter in the brain. For example, it is involved in synaptic plasticity, axonal development and neurodegenerative diseases. Glutamate dysregulation may induce excitotoxicity, which is closely associated with multiple psychiatric and cognitive disorders [1-4]. Although microdialysis methods have been used over two decades to detect extracellular glutamate levels in the brains of conscious animals, various authors have provided evidence that the glutamate detected by this method is not or only partly derived from synaptic transmission. Therefore an easy, quick and direct detection system of glutamate from synaptic origin is of great importance.

Over the last decade significant progress has been made in the development of immobilized enzyme-based electrochemical biosensors (diameter: 10 to 25 μm) for monitoring extracellular glutamate. Various research groups have published different types of enzyme-based electrochemical glutamate sensors that have been applied *in vivo* (Table 1). Some research groups reported the application of their glutamate sensors in freely moving animals [5, 6].

**Figure 1.**
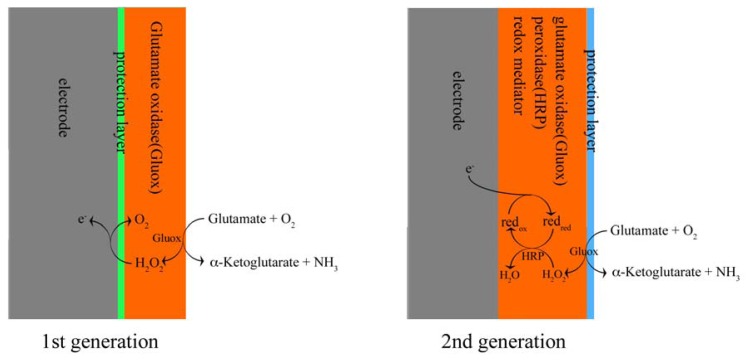
Working mechanisms for first and second generation of glutamate sensors.

These different types of amperometric glutamate sensors are based on working principles that can be divided into two main groups, according to their redox reactions taking place on the electrodes and the different electron transfer mechanisms. Here we classify them into “first” and “second” generation glutamate sensors. (Figure 1)

First generation enzyme-based electrochemical sensors were developed during the 1960s and early 1970s [13-15]. Detection is based on one-step redox reaction catalyzed by a specific oxidase, for example glucose oxidase or glutamate oxidase, where after the O_2_ consumed or H_2_O_2_ produced during this oxidation is detected directly by the electrodes.

Second generation sensors were developed in the 80s' and 90s' [16, 17] and possess a more complicated structure. A two-step redox reaction is involved in this type of sensors. As with first generation sensors, a certain biological substance is first oxidized by O_2_ on the surface of the sensors, and H_2_O_2_ is produced. The H_2_O_2_ is further reduced by a redox mediator. In this step, a second redox enzyme is required, normally peroxidase. Therefore in second generation sensors reduction/oxidation of the redox mediator is used to detect the analyte of interest. In general, these redox reactions are performed at a lower potential, which result in sensors with less sensitivity for interfering compounds.

Both generations of sensors have advantages and disadvantages. As we are very interested in the practical application of immobilized enzyme-based electrochemical sensors to monitor glutamate levels *in vivo*, both generations of sensors are applied in our laboratory [18]. Here we compare different properties of these two types of sensors, including sensitivity, specificity, reproducibility and performance *in vivo*, in order to identify the most suitable type of sensors for further experiments.

## 2. Structure, material, binding system of sensors and working mechanisms

First generation glutamate sensors are relatively simple instruments (Figure 1). They consist of an electrode covered by a protection layer and an enzyme layer. Normally the electrode is a platinum cylinder or disk. In the Gerhardt group screen printed platinum recording site (15 × 333 μm, S2 type) based on ceramic substrate [6, 7] was used as microelectrode. Carbon fiber is also often used as electrode material for first generation sensors, but compared with platinum it is less sensitive: the glutamate detection limit for the carbon fiber electrode (CFE) is 2.5 μM [10], and 20 nM for the platinum electrode [19]. We used the platinum cylinder of 25 μm in diameter and 500±50 μm in length for our first generation sensors.

The surface of the electrode material is covered by protection and enzyme layers. The protection layer eliminates non-specific signals by preventing interference compounds to reach the bare electrode. Different research groups use different orders of protection and enzyme layers: some research groups apply the protection layer under the enzyme layer, while other groups coat the protection layer on the outside of the enzyme layer. There are quite a few studies about the selection of the material for this protection layer, which will be later discussed in detail.

**Figure 2.**
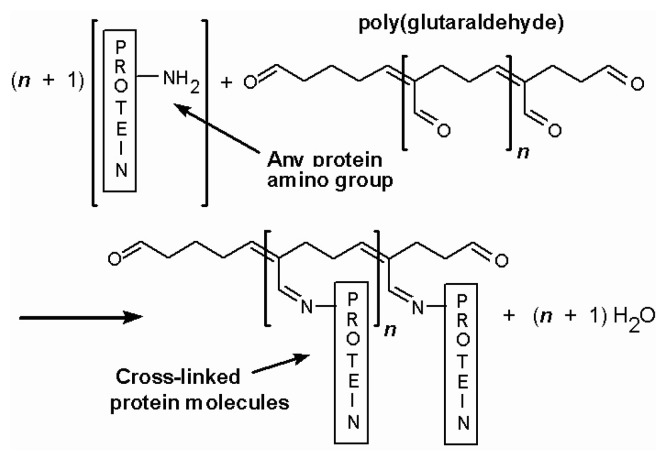
Cross-link reaction between glutaraldehyde and proteins.

The enzyme layer is normally composed of glutamate oxidase, a cross linker and a protein stabilizer. First generation sensors mostly use glutaraldehyde as the cross linker (Figure 2) and bovine serum albumin as the enzyme stabilizer. The amount of the most important composite of the sensors, glutamate oxidase, is quite high, ranging from 100 to 200 U/mL. In our laboratory, we find that 200 U/mL of glutamate oxidase produces the most stable sensors. The enzyme layer may also be applied in a number ways, such as manual coating, drop coating, dip coating and spray coating. Manual coating with microdrops (∼ 1 μL) of enzyme mixture is predominantly used. After coating, a very thin yellow transparent layer of enzyme can be seen under a microscope.

On first generation sensors, glutamate is oxidized with glutamate oxidase and produces α-ketoglutarate and H_2_O_2_. At high potential, for example +700 mV for platinum, the electrode gains electrons from H_2_O_2_ and finally converts H_2_O_2_ into O_2_. In a few cases, consumed O_2_ is detected by its reduction on the electrode at very low potential, for example –650 mV [20].

Second generation glutamate sensors are composed of an electrode, an enzyme layer and an outside protection layer. CFE, platinum and screen printed graphite-silver electrodes are often used in these sensors [18, 21, 22].

**Figure 4.**
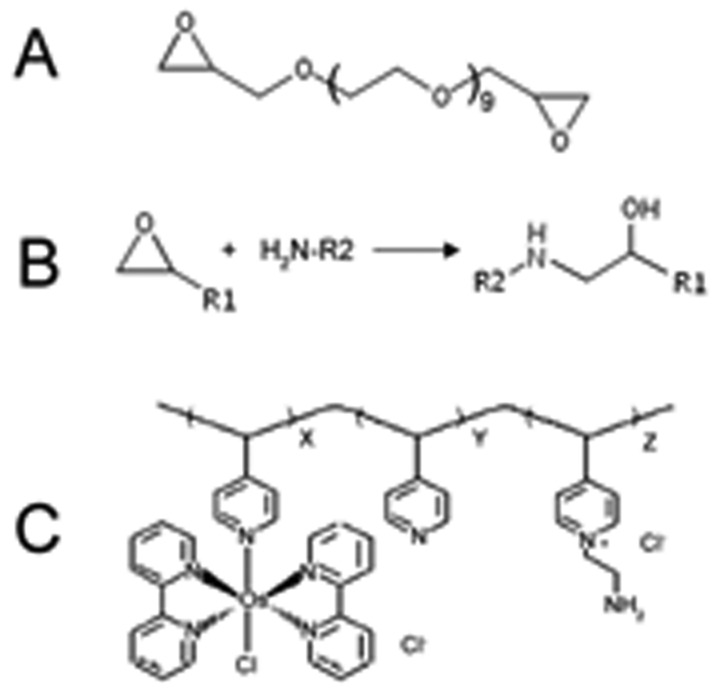
Compositions of the hydrogel-coated glutamate microsensor. (A) Poly(ethylene glycol) diglycidylether (PEDGE), the cross-linker that connects the enzymes to the osmium redox polymer. (B) Cross-link reaction between enzymes (protein) and PEDGE. (C) Osmium redox polymer; ratio of side groups x:y:z is 1.0:4.0:1.2

The composition of the enzyme layer of the second generation sensors is more complicated, compared with that of the first generation ones since it is composed of glutamate oxidase (or glutamate dehydrogenase + NADH + NADH redox mediator), peroxidase, a cross linker, a redox mediator and sometimes ascorbic acid oxidase to prevent ascorbic acid (AA) interference. The redox mediator has to meet certain criteria. Ideally, it should show reversible heterogeneous kinetics: have stable oxidized and reduced forms, should react rapidly with the second redox enzyme – peroxidase, the potential applied for the regeneration of the oxidized mediator should be low and pH independent, the reduced form should not react with O_2_ [23] and it should be tightly anchored into the architecture of the biosensor. Soluble low-molecular weight metal complexes are mainly used for this purpose. The material of second generation electrodes also has to match with the redox mediator electrochemical property for optimizing electron transfer. For example, our cyclic voltammetric studies suggested that the CFE responds better to the oxidation-reduction of osmium complex used as the redox mediator than the platinum wire electrode (Figure 4). Therefore, attentions should be paid on the selection of materials with respect to their interactions and the efficiency of redox reactions in order to obtain better response. Since a large amount of redox mediator is involved in this enzyme layer, and it contains more composites, the thickness of the enzyme layer of second generation sensors is slightly larger than that of first generation sensors. The second generation sensors developed in our lab have an enzyme layer of about 5 μm thick.

A protection layer coats the outside of the sensors. It not only prevents non-specific electrochemical interferences reacting with redox mediators and electrodes, but also decreases biofouling of sensors. Biofouling, defined as unspecific attachment of biological material to electrode surfaces upon their exposure to brain tissue, can influence the performance of sensors, including decreasing sensitivity and increasing interference response.

**Figure 5.**
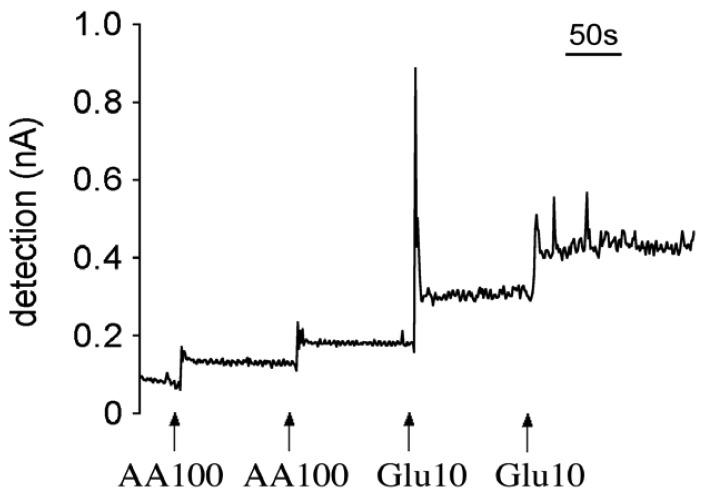
A typical raw amperometric calibration profile of a first generation glutamate microsensor at +700 mV. Calibration was performed in a stirred beaker system. Interfering compounds and analyte were added to beaker after electrical current was stabilized.

In order to reduce the detection potential, second generation sensors work with a more complicated and longer reaction chain. Different redox mediators, such as Prussian Blue [22] and osmium polymer complex [24], lead to different subtypes of second generation sensors that have different electron transfer mechanisms. Here we use a hydrogel sensor previously developed by Kulagina *et al.* [8] as an example. These sensors consist of a CFE that is coated with a redox-hydrogel. The CFE has a diameter of 10 μm and a length of 300-400 μm. The hydrogel enzyme layer contains five components: glutamate oxidase; ascorbic acid oxidase; horseradish peroxidase, the second redox enzyme; osmium redox polymer (POs-EA), which is the redox mediator, it is composed of a poly(vinylpyridine) backbone complexed with osmium (bipyridine) chloride groups and partially quarternized with ethylamine groups; and polyethylene glycol) diglycidylether (PEDGE) as the cross linker, that covalently cross-links the enzymes with POs-EA at their amino groups via a ring-opening reaction. (Figure 5) Finally, on the outside of the hydrogel layer a thin Nafion protection layer is applied to prevent biofouling.

On these hydrogel-based second generation of sensors, the glutamate is firstly converted into α-ketoglutarate under glutamate oxidase catalysis and H_2_O_2_ is produced in this step; then the H_2_O_2_ is further reduced by Os^2+^ into H_2_O under catalysis of horseradish peroxidase, the Os^2+^ is oxidized into Os^3+^ on the CFE surface at -100 mV, the CFE losses electrons and the Os^3+^ gains these electrons to convert back into Os^2+^. This way the osmium is recycled and acts as an electron transfer mediator.

I. $\text{Glutamate}+O_{2}\overset{\text{Glutamate oxidase}}{\to }\alpha -\text{ketoglutarate}+{\text{H}}_{2}{\text{O}}_{2}$
II. $2\text{O}{\text{s}}^{2+}+2{\text{H}}^{+}+{\text{H}}_{2}{\text{O}}_{2}\overset{\text{Horseradish peroxidase}}{\to }2\text{O}{\text{s}}^{3+}+{\text{H}}_{2}\text{O}$
III. 2Os^3+^ + 2e^-^ → 2Os^2+^

The working potential used here, –100 mV, is much lower when compared with that used for first generation sensors. It is a negative potential because the osmium mediators have to be present in the reduced state, Os^2+^. Therefore non-specific oxidation reactions of easily oxidizable interfering compounds are prevented.

The structure of first generation sensors is simple and clear. It is relatively easy to control the application of the layers; resulting in high reproducibility of sensitivity and specificity of the sensors. In contrast, in the case of the second generation sensors, more than two enzymes need to bind to the cross-linker which is connected to the poly(vinylpyridine) backbone. It is difficult to characterize the final concentrations of these different composites and the spatial relations between every transducer. In addition, this complicated structure is formed in one preparation step. Even with a semi-automatic dip coater, the reproducibility is quite low.

However, we have noticed a remarkable property of the second generation sensors: the concentration of glutamate oxidase in the enzyme coating mix (1.13 U/mL) is about two orders of magnitude less than that of first generation sensors. For first generation sensors, part of the glutamate oxidase might be employed to make the enzyme layer more compact, thereby acting as a “protection layer” that eliminates the passage of interfering compounds through the layers. In the second generation sensors, the competition among different enzymes to react with cross-linker limits the amount of glutamate oxidase that can be applied in the enzyme coating mixture. In addition, the reaction-chain in this type of sensors has an amplification effect. Therefore, much less glutamate oxidase is used in the second generation sensors.

## 3. Characteristics of sensors *in vitro*

The immobilized enzyme-based electrochemical biosensor is a promising tool in the measurement of extracellular glutamate *in vivo*. In order to obtain good analytical performance *in vivo* sensors should first of all meet specific requirements *in vitro*. The characteristics of different generations' biosensors *in vitro* are shortly reviewed here. To that end we have evaluated both generations of glutamate sensors in our laboratory. We determined their response time, sensitivity, linear range and specificity. These parameters were measured with the same calibration system, including a flow injection analysis system (FIA) and a potentiostat (Pinnacle Technology) with its recording and analysis software. The response time of the sensors was measured in a stirred beaker instead of with the FIA system.

### 3.1 Response time

**Figure 6.**
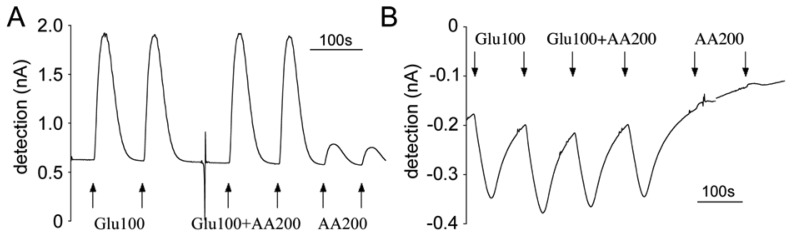
Typical raw amperometric calibration profile of the first and second generations of glutamate microsensors with FIA system at flow rate of 1 mL/min. Interfering compounds and analyte were administered in duplicate as 30 second bolus injections. (A) Calibration with the first generation sensor at +700 mV. (B) Calibration with the second generation sensor at –150 mV.

Compared with the second generation sensors, the first generation sensors are composed of a simple and thin enzyme structure, therefore the electrons generated by the redox reaction of H_2_O_2_ can rapidly reach the electrodes. We measured the response time of both generations of sensors in a stirred beaker to which glutamate solution was applied with and without interfering compounds, such as AA. Since steady-state conditions were established in this approach, we could assume that the amount of substrates diffusing into the enzyme layer closely approximated the amount that was consumed by the enzymatic reaction. Figure 6 shows a typical sensor response in the beaker calibration system. The response time for the first generation sensors was 3 to 4 seconds, while no difference was observed in the presence or absence of AA. The response time of the second generation sensors was about 9 seconds in the presence of AA and 12 minutes in the absence of AA [18]. It suggested that under *in vivo* conditions, where AA is normally present, both types of sensors can pick up glutamate signals very quickly; but the first generation sensors can respond faster than the second generation sensors.

Other sensor parameters were measured with the FIA calibration procedure. Here steady state conditions did not occur because the detection substrate would be replaced by artificial cerebrospinal fluid (aCSF), the substrate carrying buffer solution, in the flow system. However, a previous study showed that the response time to glutamate in the presence of reducing agents (AA) determined with FIA and within a stirring beaker is very similar [18].

### 3.2 Sensitivity and linearity of the glutamate response

**Figure 7.**
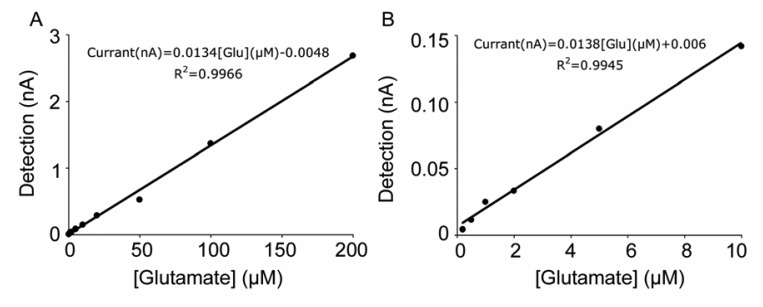
Calibration curve demonstrating the linearity of the first generation glutamate sensor. (A) Glutamate concentrations were from 0.5 to 200 μM. (B) Linear curve at low glutamate concentration.

As discussed in the section on working mechanisms, first generation sensors directly detect the oxidation of the H_2_O_2_ produced by oxidation of glutamate via glutamate oxidase immobilized on the sensors. Therefore a relatively high positive potential is required between the sensors and the reference electrodes. We use +700 mV vs. Ag/AgCl for our first generation sensors. A positive oxidation peak was observed in amperometric graphs when glutamate contacted the sensors (Figure 7A). On the contrary, the hydrogel-based second generation sensors operated at a lower negative potential of –150 mV, which is necessary for maintaining osmium in its reduced state and facilitating the reduction of Os^3+^ on the electrode. Therefore when the glutamate flow reached at the sensor, a negative reduction peak was observed (Figure 7B).

According to the available literature, first generation sensors have a wider glutamate detection range (Table 1). Our experiments with the same evaluation system confirmed this observation. Both generations of sensors showed a linear dose response to glutamate. With the first generation sensors, the detection limit was less than 0.5 μM (Figure 8B), while the second generation sensors could not generate linear amperometric values when glutamate concentration was below 5 μM [25].

We used a concentration of 100 μM glutamate to calculate the sensitivity, as this concentration is within the linear range of the method. The sensitivity of our first generation sensors was 11.9 ± 2.3 pA/μM (n = 12) glutamate delivered by the FIA system at 1 mL/min and at 37°C. The sensitivity of the second generation hydrogel sensors was 2.0 ± 0.3 pA/μM (n=23) at the same experimental condition, which was quite low compared with the first generation sensors. Taking into account the difference in the quantity of glutamate oxidase used in the enzyme coating mixtures, the structure of the second generation sensors must be considered more effective. However, overall the first generation sensors were more sensitive to glutamate.

### 3.3 Specificity

In the brain there are several ubiquitous electrochemically active substances that may react with glutamate sensors in a non-specific way because of their strong reducing character. For example, ascorbic acid (AA) and uric acid (UA) easily become oxidized on the surface of electrodes even at a low potential [26, 27]; in addition they may react with the oxidized state enzyme(s) or redox mediator (HRPox, Os^3+^ and H_2_O_2_).

The sensors' specificity was challenged with four different electrochemically active interfering substances: AA, UA, dopamine (DA) and cysteine. A semi-selective protection layer can limit the diffusion of those interfering compounds, by reducing their direct contact with the electrodes. Therefore the specificity of the first generation sensors was improved significantly by coating with a proper protection layer. The role of the protection layer will be discussed later in this review. The second generation sensors contain ascorbic acid oxidase, which can catalyze AA oxidized into dehydroascorbic acid and producing H_2_O. Moreover the second generation sensors operated at a low negative potential. As a consequence the possibility of the oxidation of a non-specific substance on the electrodes was greatly reduced, which explains the limited interference detection for both generations of sensors (Table 2).

## 4. Interference and solutions

As previously mentioned, the interference from different electrochemically active substances in the brain may lead to a decrease in the sensitivity and specificity of glutamate sensors. Most of the interference is from strong reducing agents, such as AA, UA, DA and cysteine. These compounds can be easily oxidized on the surface of the electrodes, especially in the first generation sensors, because a high potential is applied; or they could react directly with the redox enzyme (HRP_ox_), the mediator (Os^3+^) on the second generation hydrogel sensors and the metabolite (H_2_O_2_) on both generations of sensors, resulting in reducing the intermediate steps in the redox reaction(s) and interfering the amperometric detection.

Glutamate oxidase is a very selective enzyme [28]. Only one study suggested that it has a slight sensitivity for I-aspartate (0.6%) [29], therefore the interference from unwanted enzymatic reactions is not a main concern for glutamate sensors.

There are several studies on preventing the non-specific interference. One of the methods predominately used with both types of sensors is the inclusion of *background sensors*. As the term implies, background sensors are prepared in the same way as glutamate sensors but without applying glutamate oxidase. During the calibration and *in vivo* experiment, the background and glutamate sensors are placed in a proximate distance (100 to 200 μm) to insure the equality of detection environments. Thereby the non-specific signal recorded through the background sensors can be subtracted from the signal of glutamate sensors. In this way, most of the interference is filtered out. However, the background and glutamate sensors do not react always exactly the same. Strong effects from interference increase the probability that background and glutamate sensors perform differently. Therefore attention should still be paid to prevent direct reaction of interfering compounds with sensors.

Besides the use of background sensors other solutions to prevent interference vary for the different generations of sensors.

For *first generation* sensors, a semi-selective membrane is often applied to prevent interference. Different polymers have shown their excellent effect on preventing interference, including non conductive polymers such as Nafion, cellulose acetate [30] and some conductive polymers, polypyrrole, phylenediamine, polypolyaniline [31] and polythiophene [9].

Nafion is one of the first polymers applied to electrochemical biosensors [32, 33] and has been reported to have an excellent barrier effect for interferences such as ascorbate. It is a sulfonated tetrafuorethylene copolymer. Because of its tetrafuorethylene (Teflon) backbone, Nafion possesses excellent thermal and mechanical stability. The combination with sulfonic acid group results in its highly cation-conductive property. Nafion membrane coated on the electrodes can prevent interference mainly via two mechanisms: firstly its complex polymer structure (with small pores) only allows small molecules to pass through; and secondly its positive charge limits the diffusion of anionic components such as AA across the membrane. To obtain a Nafion coated surface, the electrodes are dipped in Nafion polymer solution and then dried. Previous studies have shown that different dipping times, concentration of Nafion and annealing with high temperature can influence the structure of Nafion and therefore affect the permeability and stability. Annealing Nafion provides a more crystal-like polymer structure which is less permeable [34-36]. When Nafion is applied to the first generation sensors, it is often annealed at high temperature.

Polypyrrole (PPy) is another polymer attracting considerable attention in the development of biosensors. It is a conducting polymer, therefore its electrical, electrochemical and catalytic properties can be easily controlled by the electrochemical oxidation process. Because of its polyacetylene-like structure, oxidized PPy has a very high conductivity and electrochemical redox activity even in pH-neutral solution. But over-oxidized PPy loses its electronic conductivity and becomes an ion-exchange membrane. On Pt electrodes pyrrole forms an ultra-thin film that is ion selective against anions, which means it only allows cations to pass through the film. Over-oxidized PPy coating can suppress effectively anionic AA but not cationic dopamine [37]. To obtain an over-oxidized PPy layer, a relatively high electrode potential is applied to control anodic oxidation. Size-selective polymer behavior was also observed by Wang *et al.* [38]. During polymer electro-deposition, a specific enzyme can be coated on the electrode together with the polymer in a one-step loading procedure: the enzyme can be mixed within the monomer pyrrole solution and co-deposited onto the electrode within the electropolymerized PPy. In this way, the enzyme is tightly entrapped in the polymer structure and the amount of enzyme and thickness of the polymer film can be well controlled [39-41]. However, this one-step loading procedure requires high concentrations of monomer and enzyme; therefore it is not an economic method to immobilize expensive glutamate oxidase on the electrode.

Electrically oxidized *o*-phenylenediamine (PD) polymer has also been applied to biosensors since the early 1990s to prevent interferences [42, 43]. The physical and electrochemical properties of the oxidized polyphylenediamine (PPD) film are independent of duration and potential of electropolymerization. In a conventional aqueous solution, a conductive film is initially formed and on top of this a non-conductive film forms which eventually insulates the electrode [44]. A recent study has shown that PPD films deposited from the hexagonal liquid crystalline phase demonstrated cation-selective permeability, which could exclude anion-like interfering substances [45]. A one-step loading procedure was used to coat the electrode with enzyme and PPD as well. The applied layer was 10-15 nm thick, which allowed fast response time [42, 46].

For *second generation* sensors, the techniques used to reduce interference are different from those developed for the first generation sensors. One approach involves the use of redox mediators to reduce the required potential for the H_2_O_2_ oxidation. Commonly used mediators includes ferrocene and its derivatives [16, 47, 48], ferrocyanide [49], quinone derivatives [50], Prussian Blue [22, 51] and osmium complex [52]. All of these redox mediators have a lower electron accept potential than the electrode potentials required for the oxidation of H_2_O_2_. The H_2_O_2_ produced by the first redox reaction is in turn reduced by the mediator to H_2_O and finally the mediator is reduced on the electrodes. This is the reason that the biosensors composed with a redox mediator apply a very low electrode potential when compared to the first generation sensors. In this way the electrochemical interference is limited. In Table 3, some mediators are listed, along with their potentials used in different enzymatic amperometric biosensors.

Another method used in second generation sensors to reduce the interference from AA is to apply ascorbate oxidase on the electrodes [8, 57]. Since AA is oxidized into dehydroascorbate and H_2_O -instead of H_2_O_2_ - by ascorbate oxidase catalysis, there is much less AA available on the electrodes to be converted into electrochemical signals.

Ion selective films are also used in second generation sensors. In some cases the membrane is coated outside the enzyme layer [25, 58]. However, there are two major concerns with this outer-layer protection membrane. One is maintaining biological activity of the enzymes. In order to avoid the damage of enzyme proteins, the film coating procedure of the second generation sensors is different from that of the first generation sensors. For example, when Nafion is coated on the sensors, it is impossible to heat them at high temperature because that would destroy the activity of the enzymes. In turn, it takes longer to completely evaporate the organic vehicle solution and the membrane is more permeable to the interfering molecules. Furthermore, since Nafion is acid, it can influence the activity of the coated enzyme layer. The second concern is that the diffusion of the substrate of interest – in this case glutamate – should not be suppressed by the protection membrane. When Nafion is applied outside the enzyme layer, diluted Nafion solution is used to coat the second generation sensors without annealing, in order to obtain a thin layer which has small effect on the diffusion of glutamate into the enzyme layer. Previous study [24] showed that with an outside Nafion layer, the interference by AA decreased from 21.7 ± 3.6 % to 14.7 ± 12.8 %. However, the sensitivity of hydrogel sensors reduced from 8.5 ± 1.7 pA/μM to 4.9 ± 1.6 pA/μM as well. Combining the effects on the sensitivity and interference suppression, the outside protection layer of the second generation- hydrogel sensors is not as effective as the protection layer of the first generation sensors.

## 5. *In vivo* detection of glutamate

The final aim of biosensor design is to detect the basal level and changes of the extracellular glutamate derived from synaptic processes in the brain of freely moving animals in ‘real-time’. When ‘real time’ detection is applied to behaving animals, the relation between glutamate transmission and behavior can be analyzed in more detail. Moreover, applied in pathological animal models, the sensors can help to select and evaluate different therapeutic strategies on regulation of extracellular glutamate concentrations.

At the time of writing there is a considerable amount of published research on the development of glutamate sensors, but most of these studies have focused on improving the performance of glutamate sensors *in vitro*. Only a few research groups have successfully applied glutamate sensors in the brain. (Table 1)

### 5.1 First generation sensors

Two types of first generation sensors are now commercially available for *in vivo* research. The first type, developed by Pinnacle Technology [59], is based on glutamate oxidase wired on a platinum-needle that is covered by protection layer(s) to exclude interferences. These sensors consist of a platinum–iridium electrode (∼170 μm in diameter and 1 mm in length). To reject interferences the sensors are firstly coated with Nafion and cellulose acetate. Next glutamate oxidase is applied. To prevent interference with AA, ascorbic acid oxidase is included in the enzyme mixture. The application potential is + 0.6 V vs. Ag/AgCl. The Pinnacle sensors are able to detect glutamate in the brain of anesthetized rats and in muscle [60]. The sensors are also designed to detect changes of extracellular glutamate level in the brain of freely moving rats [12, 61]. To that end the output of these sensors are connected to a wireless head-mounted potentiostat, which avoids the limitations on animal movements by the wire connection and diminishes the damage of the connection cable by animals. However, the study on which the Pinnacle sensors are based [12] reported a pronounced biofouling effect: the sensitivity to glutamate decreased by an average of 51.3 ± 4.7% after exposure to brain tissue, which is due to the absorption of endogenous species on the sensor surface. Moreover the method reported did not include application of background sensors. Another disadvantage of the Pinnacle sensors is their size: they are not much smaller than the smallest microdialysis probe (∼200 μm in diameter). In addition, there is little information about these sensors regarding the specificity of the detected basal level of extracellular glutamate, nor is there any evidence of response to the application of the sodium-channel blocker tetrodotoxin (TTX).

A different approach was followed with commercially available sensors that were recently developed by the Gerhardt group. These glutamate sensors consist of a ceramic-based microelectrode array with multiple Pt recording sites. The ceramic base is a triangle shape of approximate 1 cm long and tapered from 1 mm to a tip of 150 μm × 125 μm with recording sites 100 μm from the tip [7]. The Pt recording sites (50 × 150 ∼ 15 × 333 μm^2^) are first coated with Nafion, which electrostatically repels anionic interferences such as AA and 3,4-dihydroxyphenylacetic acid (DOPAC). Thereafter, the sites are coated with a protein matrix layer containing GluOx. The working potential is + 0.7 V vs. Ag/AgCl. According to its working mechanism these sensors belong to the first generation. By coating different recording sites on the same sensors with glutamate oxidase or matrix [62-64], interfering signals can be subtracted in order to produce a more specific glutamate signal [62]. This principle is called “self-referencing” and might be more specific than the traditional use of background sensors. It has been shown that these sensors were capable of detecting the fast increase of glutamate release evoked by potassium on a sub-second scale; in addition the sensors were also used to investigate the uptake speed of locally injected exogenous glutamate [65, 66].

Two studies, one in anesthetized rat brain and the other one in freely moving mouse brain have shown that the glutamate signal of the Gerhardt sensors responded to local application to TTX. In anesthetized rat, the average TTX-dependent changes in glutamate concentration were 1.9 ± 0.7 μM and 2.0 ± 0.8 μM in striatum and frontal cortex respectively, considering basal glutamate concentrations of 1.8 ± 0.3 μM and 1.8 ± 0.4 μM in striatum and frontal cortex. In other words, an approximately 100% decrease was observed for the detectable glutamate by 100 nL of 100 μM TTX local application in anesthetized rat [7]. However, in the striatum of conscious mouse, less than 20% of the basal glutamate concentration responded to 1.0 μL of 1 μM TTX application [5]. More research regarding the origin of the recorded glutamate by these sensors is therefore needed.

Several studies have shown the application of these sensors in brains of different species, including rat, mouse and monkey [6, 5, 67] at different ages [65]. In addition, the sensors were also used in transgenic mice to study the effect of partially or completely knockout dopamine D4 receptor on glutamate clearance in the brain [68].

A great advantage of these sensors is their capability to detect extracellular glutamate in freely moving mouse and rat [6, 5]. Moreover, this method has been reported to be ready for application in humans [67].

Recently, Rahman *et al.* [9] have developed Pt needle-type (25 μm in diameter and ∼ 300 μm long) glutamate sensors. The design is based on the covalent immobilization of glutamate oxidase onto a conducting polymer (CP) of 5,2′:5′,2″-terthiophene-3′-carboxylic acid on the electrodes. Co-immobilizing ascorbate oxidase and coating the sensor surface with a cationic polymer, polyethyleneimine minimized the endogenous reducing agent interference. These biosensors efficiently detected glutamate through the oxidation of enzymatically generated H_2_O_2_ at +0.45 V vs. Ag/AgCl. The basal extracellular glutamate concentration in anesthetized animals was determined to be about 2.0 ± 0.5 μM in rat striatum based on the post *in vivo* calibration. The biofouling was about 29%. These sensors detected an increase of extracellular glutamate after repeated injections of cocaine. Unfortunately, to date there have been no further *in vivo* pharmacological evaluations (such as TTX-dependency) reported for these sensors.

### 5.2 Second generation sensors

The sensor designed by Kulagina *et al.* [8] was the first second generation type to be used *in vivo*. These microsensors were composed of a carbon fiber (10 μm in diameter and 300-400 μm long), which was coated with a redox mediator wired to peroxidase, glutamate oxidase and ascorbate oxidase by a cross-linker. A thin Nafion film was used as an outside protection layer. The redox mediator allowed a very low detection potential, –0.1 V vs. Ag/AgCl. The sensors were applied in brains of anesthetized rats. In this work, high basal levels of glutamate were reported: 29 ± 9.0 μM in striatum. The synaptic origin of the recorded glutamate was demonstrated by local infusion of 200 nL of TTX 100 μM, which induced a 25-85 % decrease in glutamate when compared with basal levels. A fouling effect was reported after *in vivo* experiments: the sensitivity of the sensors decreased by 50-65 %. To correct this effect, the calculation of glutamate concentration was based on post *in vivo*.

More recently, in our laboratory Oldenziel *et al.* have improved the performance and reproducibility of the second generation hydrogel sensors by optimizing the composition [24], using an automatic dip coater machine [25] and purifying ascorbate oxidase [57]. These sensors were constructed by coating a CFE (10 μm diameter; 300-500 μm long) with a five-component redox hydrogel, in which L-glutamate oxidase, horseradish peroxidase and ascorbate oxidase were wired via PEDGE to an osmium-containing redox polymer.

These sensors could detect extracellular glutamate in various pharmacological studies. They demonstrated fast changes of glutamate concentration in anesthetized rat brain under different pharmacological conditions, including local application of exogenous glutamate, KCl, glutamate uptake blocker (TBOA) [18, 11]. In particular, the glutamate concentration decreased by about 90% of basal level immediately after the TTX application. This suggested that the glutamate measured by these hydrogel sensors was derived predominantly from exocytotic release.

One of the major disadvantages of these second generation sensors was their sensitivity to the interference by AA. An optimal inclusion of ascorbic oxidase activity in the hydrogel was required to prevent this interference. Moreover, because of the small size of the carbon fiber and the variability inherent to the dipping procedure, it was difficult to fabricate sensors in a reproducible way. The results of *in vivo* studies, including the TTX-dependency, could not always be replicated. Moreover, because of the fragility of the carbon fiber, it would be difficult to develop a model for freely moving animals.

### 5.3. The extracellular concentration of glutamate

The various *in vivo* studies using first and second generation glutamate sensors have produced different values of basal extracellular glutamate concentrations. The basal levels of glutamate detected by the sensors produced by Oldenziel *et al.* varied between 18.2 ± 9.3 μM and 23.6 ± 5.3 μM in striatum. This result is accordance with the observation by Kulagina *et al.*: 29 ± 9.0 μM in striatum. Lower levels were reported by Rahman *et al.* [9]: 2.0 ± 0.5 μM in rat striatum. The basal values reported by the Gerhardt group displayed a large variation: 2-40 μM. This is partly explained by different anesthesia depth during measurement: the deeper the level of anesthesia, the lower the current output of the sensors [11]. In general microdialysis studies have reported somewhat lower basal values (1-5 μM). Note that in all the experiments with the second generation glutamate sensors, post *in vivo* calibration was taken to correct the biofouling effect on sensors: their sensitivity decreased considerably. While for the first generation sensors from Pinnacle Technology and the Gerhardt group, pre-*in vivo* calibration was used to calculate the glutamate concentrations. Therefore, the glutamate basal levels observed in these first generation sensors could have been underestimated.

Kulagina *et al.* also suggested that the small size of the hydrogel based glutamate sensors causes less damage to the adjacent tissue, implicating that the implantation had less influence on neuronal activity; therefore the higher levels of glutamate might be explained by a more pronounced neuronal activity in the vicinity of the microsensors. In this regard it is of interest to compare the damage caused by the different sensors or microdialysis probes. Borland *et al.* [69] provided evidence that dopamine release was disrupted within 220 μm from microdialysis probes in tissue, whereas the damage caused by the hydrogel-based carbon fiber sensors was only seen within an area of 3 μm in diameter around the fibers. The damage caused by the Gerhardt sensors was substantial and seen at distances of 50 -100 μm around the sensors [6]. Therefore the significance and origin of basal glutamate levels detected by the currently available glutamate sensors need further investigation.

## 6. Discussion

### 6.1. Sensitivity of the sensors

It appeared that the average sensitivity of the first generation sensors is about five times higher than that of the second generation sensors. However the efficiency of glutamate catalysis in the second generation sensors is apparently much higher, since we noticed that these sensors require two orders of magnitude less glutamate oxidase. Although there is an amplification effect in the reaction chain of the second generation sensors, it is likely that the amount of immobilized glutamate oxidase in the first generation sensors is much more than it needs for catalyzing glutamate. Improving the structure of matrix on the electrode of first generation sensors (for example by optimizing the spatial arrangement of enzyme with cross-linker and stabilizer to obtain a better exposition of the active site, in order to increase the efficiency of glutamate oxidase within the enzyme layer) would probably be helpful for increasing the sensitivity of sensors and economizing the expensive enzyme.

### 6.2. Speed and selectivity of the sensors

The initial objective of glutamate sensor design – to detect rapid changes in synaptic glutamate concentrations in the brain of freely moving animals – has not yet been fully accomplished with the existing sensors. Comparing these two types of sensors, the first generation sensors possess a simpler structure than the second generation sensors and therefore they respond faster. One of the examples are the commercially available sensors developed by the Gerhardt group, which detect extracellular glutamate levels in the brain of freely moving animals in sub-second scale.

For studies into the real-time regulation of glutamate in the brain two key challenges remain: improving response time and addressing interference. Since glutamate transport cycle by glial cells is less than 100 ms, more accurate detection is highly desirable. The response time of the sensors depends mostly on the kinetics of the enzymes and diffusion speed of H_2_O_2_ to the electrode. Moreover, biofouling can induce an increase in response time and interference during long term implantation of sensors *in vivo*. Hence there is ongoing need to optimize the construction of glutamate amperometric sensors, with a particular focus on the construction of the enzyme application matrix and protection layer. Improving the enzyme efficiency may also help to increase the response speed of sensors. Protection membrane that is highly selective for glutamate or H_2_O_2_ (depending on protection layer out- or inside the enzyme layer) and that prevents interfering substances to reach the bare electrode is a critical requirement for the sensors. At the same time, the protection and enzyme layers should allow a fast diffusion of glutamate and H_2_O_2_ molecules. During long term implantation, the protective effect of the protection layer is even more important since biofouling needs to be prevented. An alternative could be using sensors with a guide system, as in the microdialysis technique, to overcome biofouling. In addition, ongoing development of more precise (for example, smaller time scaled) recording equipment might be helpful to detect glutamate levels with higher temporal resolution.

Other improvements might also be considered. A smoother electrode surface may cause less damage to brain tissue and decrease the interaction between electrode surface and biological elements such as cells and blood vessels.

### 6.3. Possible synaptic origin of recorded glutamate

In spite of their relatively lower sensitivity, the second generation sensors detect higher concentration of glutamate *in vivo*. This observation might involve a greater contribution of glutamate derived from synaptic sources, which might be explained by the fact that the smaller size of the second generation electrodes cause less damage compared with first generation sensors. But the size of sensors is still much larger than that of the synaptic cleft. Due to the fact that the concentration of extra-synaptic glutamate decreases dramatically because of glial glutamate uptake, even with proximate distances between some synaptic clefts and sensors, glutamate will probably distribute heterogeneously on the surface of sensors during *in vivo* detection. Since the calculation of glutamate concentration is based on the signal recorded through the whole surface, there could be an average effect on the detected glutamate concentration *in vivo*, which is much lower than the glutamate concentration in synaptic clefts.

The question whether neuron-released glutamate can reach extra-synaptic sources is still a matter of debate, although various authors have provided evidence that under certain conditions (for example during behavioral activation) impulse-flow dependent glutamate can reach extra-synaptic sites. It still remains unclear to what extent the various sensors are able to discriminate between synaptic and glial glutamate. With the second generation sensors a certain TTX-dependency was reported, but the reproducibility of this type of sensors remains an issue. According to the publications of the Gerhardt group, the inhibition effect of TTX (sodium channel blocker, non-specific neurotransmission blocker) on the detected extracellular glutamate levels varies between different types of animals and anesthetized states. Without the availability of a specific synaptic glutamate release blocking agent, it will be difficult to identify the origins of detected glutamate.

## Figures and Tables

**Figure 3.**
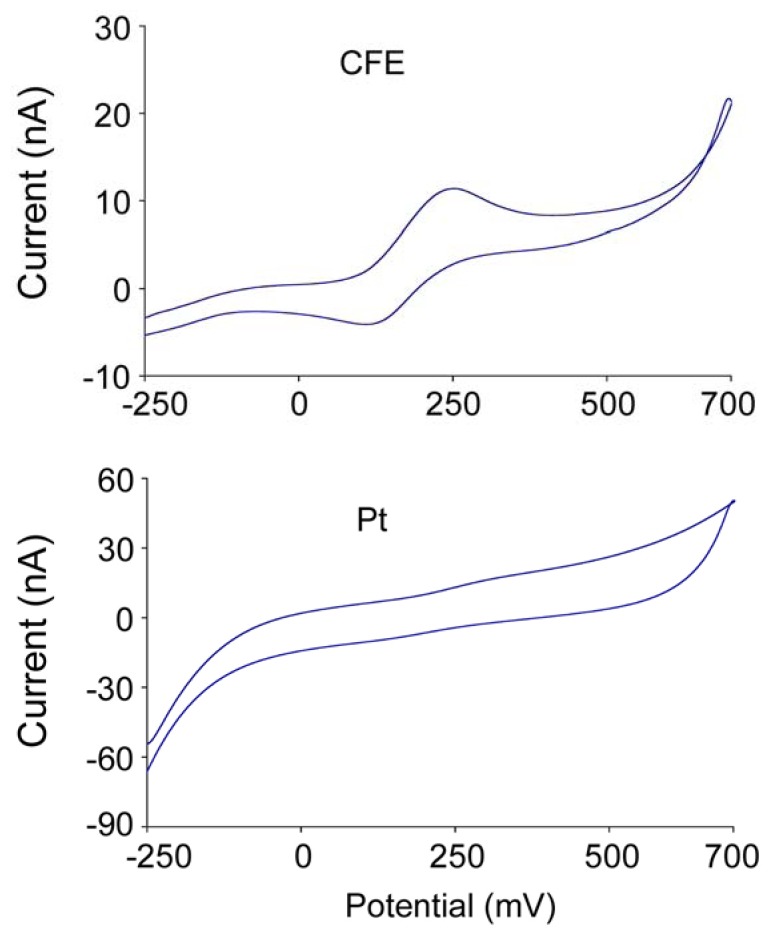
Cyclic voltammogram recorded for carbon fiber and Pt electrodes vs. Ag/Ag/Cl in 1 mg/mL osmium redox polymer solution at 100 mV/s displayed on two current density scales. The scan for the CFE was in a smaller magnitude and showed the oxidation/reduction peaks; while for the Pt electrode, there were no detectable oxidation/reduction peaks observed.

**Table 1.** Different types of enzyme-based amperometric glutamate microbiosensor. CFE, carbon fiber electrode; CP, contact polymer; Ru, ruthenium; SCE, Saturated calomel electrode; Ir, iridium.

**Research group**	**Characteristics of sensor*in vitro***						**Generation**	**Reference**

	**Electrode**	**Surface (mm^2^)**	**Potential (mV) vs. Ag/AgCl**	**Response time (s)**	**Sensitivity (nA/μM)**	**glutamate detection limit(μM)**		

Gerhardt	Pt site	0.005	+700	∼ 1	0.016 ± 0.001	1.82 ±0.17	1st	[7]
Michael	CFE	0.0095 ∼0.0126	-100	20 ∼ 40	0.0034 ± 0.001	1∼3	2nd	[8]
Shim	CP coated Pt cylinder	0.024	+450	∼ 10	14.0 ± 0.2	0.1± 0.03	1st	[9]
Soldatkin	Ru coated CFE	0.048	+400	-	0.029	2.5	1st	[10]
Westerink	CFE	0.0095 ∼0.016	-150	∼ 8	0.0055 ± 0.00007	5	2nd	[11]
Wilson	Pt/Ir	0.183	+600	∼ 1	0.1	2	1st	[12]

**Table 2.** Selectivity of both generations of sensors. The values were expressed as percentage of the current generated with 100 μM glutamate.

		**AA 200 μM**	**UA 50 μM**	**dopamine 5 μM**	**cysteine 5 μM**
1st generation	+ Glu 100μM	122±4 (n=4)	101±5 (n=4)	116±4 (n=4)	117±9 (n=4)
	-	17±2 (n=4)	4±0 (n=4)	23±4 (n=4)	3±0 (n=4)
2nd generation	+ Glu 100μM	78±5 (n=19)	72±27 (n=14)	98±6 (n=9)	108±11 (n=9)
	-	3±3 (n=19)	0±0 (n=14)	12±7 (n=9)	18±10 (n=9)

**Table 3.** Enzymatic biosensors with different redox mediators and their application potentials.

**Enzymatic biosensor**	**Electrode**	**Mediator**	**Redox potential**	**Reference**
Glucose oxidase	sono-gel carbon composite (SCC) electrode	ferrocene	0.30 V vs. SCE	[53]
Glucose oxidase	Glass carbon electrode	Poly(*m*-aminoanilino methylferrocene)	-0.05 V vs. Ag/AgCl	[54]
Glucose oxidase	Pt	polyvinylferrocenium	0.30 V vs. SCE	[55]
Glutamate oxidase	Pt	Prussian Blue	0.0 V vs. Ag/AgCl	[51]
Glucose oxidase	Carbon rotating disk electrodes	[Os(bpy)_2_Cl^]+1/+2^	0.40 V vs. Ag/AgCl	[56]
Glutamate oxidase	CFE	[Os(bpy)_2_Cl^]+2/+3^	-0.15 V vs. Ag/AgCl	[18]
